# LasR Variant Cystic Fibrosis Isolates Reveal an Adaptable Quorum-Sensing Hierarchy in *Pseudomonas aeruginosa*

**DOI:** 10.1128/mBio.01513-16

**Published:** 2016-10-04

**Authors:** John B. Feltner, Daniel J. Wolter, Christopher E. Pope, Marie-Christine Groleau, Nicole E. Smalley, E. Peter Greenberg, Nicole Mayer-Hamblett, Jane Burns, Eric Déziel, Lucas R. Hoffman, Ajai A. Dandekar

**Affiliations:** a Department of Medicine, University of Washington, Seattle, Washington, USA; b Department of Pediatrics, University of Washington, Seattle, Washington, USA; c Department of Microbiology, University of Washington, Seattle, Washington, USA; d Seattle Children’s Hospital, Seattle, Washington, USA; e INRS-Institut Armand-Frappier, Laval, Québec, Canada

## Abstract

Chronic *Pseudomonas aeruginosa* infections cause significant morbidity in patients with cystic fibrosis (CF). Over years to decades, *P. aeruginosa* adapts genetically as it establishes chronic lung infections. Nonsynonymous mutations in *lasR*, the quorum-sensing (QS) master regulator, are common in CF. In laboratory strains of *P. aeruginosa*, LasR activates transcription of dozens of genes, including that for another QS regulator, RhlR. Despite the frequency with which *lasR* coding variants have been reported to occur in *P. aeruginosa* CF isolates, little is known about their consequences for QS. We sequenced *lasR* from 2,583 *P. aeruginosa* CF isolates. The *lasR* sequences of 580 isolates (22%) coded for polypeptides that differed from the conserved LasR polypeptides of well-studied laboratory strains. This collection included 173 unique *lasR* coding variants, 116 of which were either missense or nonsense mutations. We studied 31 of these variants. About one-sixth of the variant LasR proteins were functional, including 3 with nonsense mutations, and in some LasR-null isolates, genes that are LasR dependent in laboratory strains were nonetheless expressed. Furthermore, about half of the LasR-null isolates retained RhlR activity. Therefore, in some CF isolates the QS hierarchy is altered such that RhlR quorum sensing is independent of LasR regulation. Our analysis challenges the view that QS-silent *P. aeruginosa* is selected during the course of a chronic CF lung infection. Rather, some *lasR* sequence variants retain functionality, and many employ an alternate QS strategy involving RhlR.

## IMPORTANCE

Chronic *Pseudomonas aeruginosa* infections, such as those in patients with the genetic disease cystic fibrosis, are notable in that mutants with defects in the quorum-sensing transcription factor LasR frequently arise. In laboratory strains of *P. aeruginosa*, quorum sensing activates transcription of dozens of genes, many of which encode virulence factors, such as secreted proteases and hydrogen cyanide synthases. In well-studied laboratory strains, LasR-null mutants have a quorum-sensing-deficient phenotype. Therefore, the presence of LasR variants in chronic infections has been interpreted to indicate that quorum-sensing-regulated products are not important for those infections. We report that some *P. aeruginosa* LasR variant clinical isolates are not LasR-null mutants, and others have uncoupled a second quorum-sensing system, the RhlR system, from LasR regulation. In these uncoupled isolates, RhlR independently activates at least some quorum-sensing-dependent genes. Our findings suggest that quorum sensing plays a role in chronic *P. aeruginosa* infections, despite the emergence of LasR coding variants.

## INTRODUCTION

Individuals with the genetic disease cystic fibrosis (CF) are predisposed to chronic lung infections with *Pseudomonas aeruginosa*, which is a significant cause of morbidity and mortality for patients with this disease (1, 2). Chronic infection-causing *P. aeruginosa* CF lineages often acquire a series of characteristic mutations (3, 4) which are adaptive to the CF lung environment. These mutations result in phenotypes that include, for instance, lack of motility and overproduction of the extracellular polysaccharide alginate. Evidence indicates that the CF lung environment also selects for *P. aeruginosa* with null mutations in the quorum-sensing (QS) transcription factor LasR (3).

*P. aeruginosa* QS is mediated in part by production of a signal, *N*-3-oxo-dodecanoyl-homoserine lactone (3OC_12_-HSL), by the LasI enzyme (5). This signal diffuses into and out of cells and binds to LasR, a member of the LuxR family of quorum-sensing transcription factors. At sufficient 3OC_12_-HSL concentrations, LasR activates the transcription of numerous target genes. Most of what is known of *P. aeruginosa* QS has comes from studying well-defined laboratory strains, in which the QS regulon encompasses over 300 genes (6, 7). One LasR-regulated gene encodes another QS transcription factor, RhlR, which responds to *N*-butanoyl-HSL (C_4_-HSL) produced by RhlI (8, 9). LasR- and RhlR-regulated genes include many that encode virulence factors (10), and LasR mutant laboratory strains show impaired virulence in animal models (11, 12). Key quorum-sensing-regulated factors include secreted virulence products, such as the protease elastase, hydrogen cyanide, and phenazines (10, 13). In addition to these acyl homoserine lactone (AHL)-mediated systems, there is another QS system in *P. aeruginosa* mediated by 2-alkyl-quinolones (14, 15). This other QS system involves binding of 2-heptyl-3-hydroxy-4-quinolone (*Pseudomonas* quinolone signal [PQS]) or its biosynthetic precursor, 2-heptyl-4-quinolone (HHQ), to the transcriptional regulator PqsR (also known as MvfR) (16, 17).

*P. aeruginosa* isolates from chronically infected CF patient lungs frequently contain mutations in *lasR* (3, 4, 18, 19) that are rare among isolates from acute infections (3, 18). Mutant *lasR* strain frequencies have been reported to be greater than 50% in some patients (3, 19), indicating strong selective pressure against LasR activity. Although *P. aeruginosa* lasR mutants are common in CF, the phenotypes of these mutants are diverse, unlike those exhibited by *lasR* mutant strains derived from the common laboratory strain PAO1 (19). Nevertheless, a few phenotypic characteristics, like colony lysis and sheen, have been associated with CF clinical *lasR* mutant strains (20). However, little is known about what factors in the CF lung might select for *lasR* mutants, or whether there are LasR-regulated *P. aeruginosa* activities that account for this selection. Understanding the evolution of *P. aeruginosa* QS in the CF lung has significant implications for ongoing efforts to develop anti-QS-based therapies (21).

Several hypotheses have been proposed for the relatively high incidence of LasR mutants in CF infections. For example, LasR mutant CF isolates (but not LasR mutants of PAO1) exhibit a small growth advantage on phenylalanine as the sole source of carbon and energy and also under high-nitrate conditions (4, 22). LasR mutants have also been proposed to be social cheaters that exploit shared QS products without incurring the metabolic cost of their production (23, 24). However, one study in a porcine infection model indicated that LasR mutants did not behave as cheaters, but rather were adapted for growth in the lung (25). *lasR* coding variations imply a loss of LasR function and, therefore, defects in many QS-regulated virulence functions (10, 26). To investigate the selective advantages favoring LasR mutants in CF lungs and the consequences of these mutations, we characterized the QS systems in a large collection of CF-derived *P. aeruginosa* isolates.

## RESULTS

### *P. aeruginosa* isolates from CF secretions harbor a wide diversity of *lasR* sequence variations.

We sequenced the *lasR* gene from 2,650 *P. aeruginosa* isolates from the Early *Pseudomonas* Infection Control (EPIC) study, a longitudinal study of early CF *P. aeruginosa* infection (27). We were unable to determine the *lasR* sequence of 67 of the 2,650 isolates due to PCR or sequencing failure, possibly as a result of deletions encompassing *lasR* or due to sequence changes in the primer sites. Of the remaining 2,583 isolates, the *lasR* genes of 580 isolates coded for polypeptide sequences different from those in the laboratory strains PAO1 and PA14 (Table 1; see also Table S1 in the supplemental material).

**TABLE 1:** Summary of the *lasR* variants in the EPIC *P. aeruginosa* collection

Mutation	Type of variation^a^	No. of isolateswith a mutation	No. of uniquemutations
Deletion	In frame	5	3
	Frameshift	67	21
Insertion	In frame	8	4
	Frameshift	12	9
	IS element	60	20^b^
Single-nucleotidechange	Missense	300	95
	Nonsense	128	21
Total		580	173

a Based on comparison to *P. aeruginosa* PAO1.

b Based on the site of IS element insertion within the *lasR* coding sequence.

**FIG 1:**
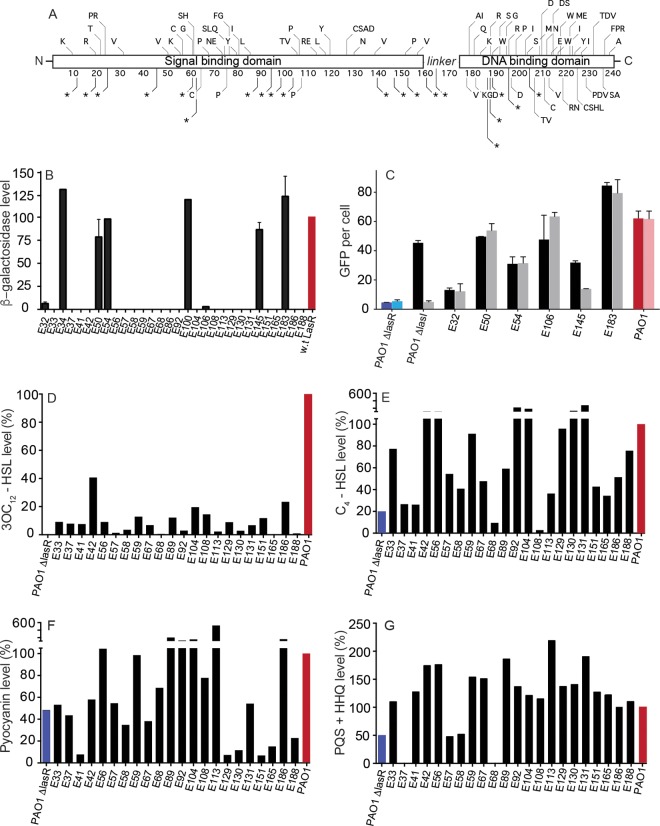
Amino acid changes, functions, and phenotypes of LasR variants. (A) Location of single-nucleotide substitutions in the EPIC isolates mapped to the LasR amino acid sequence. Each unique amino acid substitution or early termination in the collection is shown in this schematic of the polypeptide. The signal and DNA-binding domains of LasR are highlighted. *, a mutation resulting in a stop codon. Where multiple letters are present, there was more than one unique substitution within the collection at the same residue (e.g., “PR” at residue 23). (B) Activity of LasR in an *E. coli* expression system. We cloned *lasR* from the isolates and expressed the cloned gene under control of an arabinose-inducible promoter in *E. coli*, which also contained a LasR-responsive *lacZ* reporter. The *E. coli* reporters were grown in the presence of 2 µM 3OC_12_-HSL. Data are the percentage β-galactosidase activity compared to *lasR* cloned from strain PAO1 (red bar). (C) Activity of LasR in clinical isolates. We electroporated clinical isolates of *P. aeruginosa* PAO1 (red) or a PAO1 Δ*lasR* mutant (blue) with a plasmid containing a LasR-responsive GFP reporter, and we measured fluorescence in the presence (dark bars) or absence (light bars) of 3OC_12_-HSL after 18 h of growth. (D and E) Concentrations of 3OC_12_-HSL (D) and C_4_-HSL (E) after 18 h of *P. aeruginosa* growth. Strain PAO1 produced 2 µM 3OC_12_-HSL and 9.5 µM C_4_-HSL. Data are displayed as a percentage of the amount of AHL produced by PAO1 (red bar). (F) Pyocyanin production after 18 h in King’s A medium. PAO1 again was used as the reference (red bar) and produced 5.5 µg/ml pyocyanin. (G) Combined production of PQS and HHQ by the various isolates after 18 h in LB broth, normalized to production by PAO1. Error bars represent mean ± ranges for results of three individual experiments.

We identified 173 unique sequence variations among these 580 isolates (the collection included multiple isolates from individual patients). Of these, 116 were single-nucleotide differences resulting in an amino acid substitution (*n =* 95) or stop codon (*n =* 21) (Table 1). Each variant was from a single patient; by excluding nonunique variants, we did not sample a diversity of isolates from individuals, as has been done elsewhere (19). The mutations were distributed throughout the *lasR* gene, although the linker region between the 3OC_12_-binding domain and the DNA-binding domain was underrepresented (Fig. 1A).

### Some *lasR* mutations code for functional proteins. 

The observation that nonsynonymous *lasR* mutations occur frequently in CF *P. aeruginosa* isolates has generally been interpreted to suggest that this protein is inactive (26). Indeed, in our study, a substantial number of the characterized mutations were insertions or deletions predicted to eliminate LasR function (Table 1); however, this prediction may not be correct for single-nucleotide substitutions. We hypothesized that some of the 116 *P. aeruginosa* LasR variants with single-nucleotide substitutions might still respond to signal and activate quorum-regulated genes. In this study, we focused on a subset of 31 isolates that had *in vitro* growth rates and yields (based on final optical densities at 600 nm [OD_600_s]) similar to the laboratory strain PAO1. Initially, we cloned *lasR* from these 31 isolates with unique single-nucleotide substitutions into *Escherichia coli* cells containing a LasR-responsive *lacZ* reporter. We grew the recombinant *E. coli* strains with added 3OC_12_-HSL and measured β-galactosidase activity (Fig. 1B; Table 2). Eight of 31 recombinant strains showed 3OC_12_-HSL-dependent expression of β-galactosidase, and 6 exhibited activities similar to that of a recombinant strain with wild-type PAO1 *lasR.* We did not identify a constitutively active variant in this study. The 23 remaining studied isolates contained nonfunctional LasR variants and are listed in Table 2.

**TABLE 2:** Location of *lasR* sequence variations in isolates with residual LasR activity^a^

Isolate	Nucleotidechange	Amino acidchange	Relative activityin *P. aeruginosa*^b^	Relative activityin *E. coli*^c^
E32	C649T	R217W	21 ± 2	5 ± 1
E34	C292T	Q98*^d^	ND^e^	130 ± 32
E50	C564A	C188*	80 ± 1	65 ± 19
E54	C350T	P117L	50 ± 8	98 ± 25
E100	A641G	N214S	ND	119 ± 21
E106	C239T	T80I	77 ± 27	3 ± 1
E145	C219G	D73E	51 ± 2	81 ± 8
E183	C478T	Q160*	136 ± 4	138 ± 22

a LasR activity was measured in *P. aeruginosa* EPIC isolates and/or in recombinant *E. coli* containing *lasR* genes from the indicated isolates, as described in Materials and Methods. The following LasR variants showed no activity in the *E. coli* reporter assay: E33 (G61A), E37 (G588T), E41 (G31T), E42 (G671A), E56 (G338A), E57 (G307T), E58 (C692T), E59 (A625G), E67 (G179A), E68 (T227G), E89 (G543C), E92 (G490T), E104 (A532G), E108 (C355T), E113 (T55C), E129 (C149T), E130 (T662A), E131 (A580G), E151 (G199C), E165 (G703A), E186 (C314T), and E188 (T452C).

b Percent activity (± standard deviation) in comparison to the laboratory strain PAO1.

c Percent activity (± standard deviation) in comparison to *E. coli* with the *P. aeruginosa* PAO1 *lasR* gene.

d An asterisk indicates a stop codon.

e ND, not determined (unable to transform clinical isolate with plasmid).

As a complementary approach, we electrotransformed the clinical isolates themselves with a LasR-responsive *lasI-gfp* reporter plasmid. Of the cohort of LasR variants that exhibited function in the *E. coli* assay, three showed reporter activity in *P. aeruginosa* similar to or greater than the laboratory strain PAO1 (Fig. 1C; Table 2). Another three isolates had reduced but evident activity compared to PAO1; one (E145) required addition of 3OC_12_-HSL for maximal green fluorescent protein (GFP) levels. Although we are not certain of the significance of the isolate that required exogenous 3OC_12_-HSL for maximal activity, one possibility is that this variant protein has a defect for folding into the fully active form, and the presence of the signal promotes proper folding. In a mixed environment of signal producers and nonproducers, these isolates would fully activate LasR.

The positions and types of variants that resulted in functional LasR varied considerably (Table 2). Three variants (E34, E50, and E183) contained substitutions that resulted in stop codons, which presumably are suppressed by second-site mutations in the isolates. Three other variants are of special interest because of their location in *lasR*, their effects in the biological assays, or both. Isolate E32 harbors a variant *lasR* gene with a C-to-T change at nucleotide 649, coding for an arginine-to-tryptophan change in the DNA-binding domain. This LasR variant demonstrated reduced function in both the heterologous and homologous expression systems, suggesting that the amino acid change results in decreased binding to or transcription from target DNA sequences, or at least the *lasI* promoter. Isolates E106 and E145 both have variant LasRs with amino acid substitutions in residues (T80 and D73, respectively) known to form the hydrophobic pocket to which 3OC_12_-HSL binds (28). These alterations raise the possibility that mutations in the signal-binding domain can affect the affinity to the signal itself, potentially changing signal specificity or altering the quorum-sensing threshold required for populations of the isolate in question.

Of the variants with no detectable LasR activity, four contained nonsense mutations which resulted in premature termination of translation: E41 (G31T), E57 (G307T), E67 (G179A), and E92 (G490T). Just under half of the remaining mutations noted were in the DNA-binding region. These included those in E37 (G588T), E42 (G671A), E58 (C692T), E59 (A625G), E89 (G543C), E104 (A532G), E130 (T662A), E131 (A580G), and E165 (G703A). Three of these (E37 [E196D], E89 [E181D], and E131 [S194G]) harbored mutations in conserved residues of the helix-turn-helix region (29) of the protein, which most probably directly disrupt binding to target DNA. Others were in the carboxy-terminal region, a finding consistent with previous reports that the DNA-binding domain of LuxR is very sensitive to mutation: even small changes (30, 31) can result in functionless protein.

Many of the LasR-null variants that we studied carried mutations in the DNA-binding domain. Three carried mutations within the defined 3OC_12_-HSL-binding pocket (28): isolates E68 (T227G/V76G), E129 (C149T/A50V), and E186 (C314T/A105V), and these mutations presumably abrogated the ability of LasR to bind signal. Additional mutations are disruptive to conserved amino acids or motifs within the defined signal-binding region (28). These include a mutation in E56 (G338A) which results in a glutamic acid substitution of a conserved glycine between the α5 and β4 motifs, and that of E108 (C355T), which results in a histidine-to-tyrosine substitution in the β4 sheet of LasR. Finally, isolate E151 contains an alanine-to-proline substitution at position 67. This residue does not form part of the binding pocket *per se*, but four of the five immediately adjacent amino acids do: D65, G68, Y69, and A70. The substitution of a proline may alter the ability of the surrounding amino acids to form hydrogen bonds with the signal. The remaining three LasR variants in the signal-binding region included in this study, E33 (G61A/A21T), E113 (T55C/W19R), and E188 (T452C/L151P), contain nonconservative substitutions in the α2 helix (E33 and E113) or the α6 helix (E188) that are likely to disrupt the structure of LasR.

### There is no correlation between the absence of a functional LasR and a variety of quorum-regulated phenotypes.

Quorum sensing, and LasR in particular, activates transcription of dozens of genes (6). In laboratory strains, LasR-null mutants produce very little elastase, 3OC_12_-HSL, or the phenazine pyocyanin, and C_4_-HSL production is reduced (6, 32). We tested whether there was a relationship between the presence of an inactivating *lasR* mutation in the isolates and the production of the AHLs or of pyocyanin. Our analysis focused on the 23 isolates in which LasR was nonfunctional (Fig. 1B and C). We found no correlation between the presence of an inactivating *lasR* mutation and the concentrations of signals or pyocyanin after overnight growth (Fig. 1D to F). We were also unable to identify an association between the presence or absence of a functional LasR and several other phenotypes, such as motility, amino acid auxotrophy, or Congo red binding (33).

Because we had previously reported associations between *lasR* mutations and colony lysis and sheen (4, 20, 34) and with growth on certain amino acids or nitrate (4), we assessed these traits in all of the EPIC isolates that were amenable to growth under these conditions. Both phenotypes were infrequent in wild-type LasR isolates and were present more commonly in the *lasR* variants, but neither correlated strongly with LasR-inactivating mutations (see Table S2 in the supplemental material).

Pyocyanin production in the LasR-null mutants often far exceeded that in PAO1 (Fig. 1F), a previously reported finding (35–38). The LasR mutants produced substantially less 3OC_12_-HSL than wild-type laboratory strains (Fig. 1D), but nearly one-third produced more than 250 nM, which is a concentration that elicits half-maximal LasR activation of gene expression in strain PAO1 (39). Furthermore, C_4_-HSL production was on average comparable to that by wild-type laboratory strains (Fig. 1E). Some isolates produced in excess of 50 µM C_4_-HSL, a level much higher than that produced by strain PAO1.

The hyperproduction of pyocyanin and C_4_-HSL by several of these LasR-null isolates raised the possibility that production of the non-AHL QS signals PQS or HHQ could contribute to the pyocyanin production phenotype. PQS and HHQ could contribute to the phenotype through PqsR induction and activation of RhlR targets via an unresolved regulatory mechanism requiring PqsE (37, 40, 41). To address this possibility, we measured concentrations of PQS and HHQ in these isolates (Fig. 1G). Although both of these molecules were produced in abundance by some isolates (for example, E113 and E131), there was no apparent correlation with pyocyanin production (Fig. 1F); indeed, some isolates (E37 and E68) produced pyocyanin despite the virtual absence of both PQS and HHQ.

### Many LasR mutant clinical isolates remain AHL responsive through the RhlR-I system.

**FIG 2:**
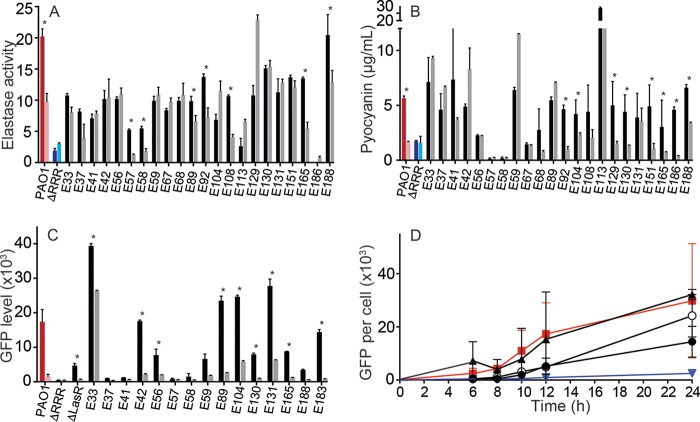
AHLs and RhlR mediate QS in some LasR-null clinical isolates. (A and B) AHL dependence of elastase and pyocyanin production. LasR-null mutants were grown in broth with (light bars) or without (dark bars) 20 µg/ml AiiA lactonase. In all cases, AiiA lactonase-grown cultures did not have measurable signals after 18 h. We measured production of elastase (A) and pyocyanin (B) from these cultures after 18 h of growth. PAO1 (red) and an AHL QS-deficient strain, mutant strain PAO1 Δ*lasR* Δ*rhlR* Δ*qscR* (ΔRRR) (blue), are shown for comparison. Elastase activity is shown in arbitrary units. (C) RhlR activity in LasR-null isolates. We electroporated AHL-responsive isolates with an RhlR-responsive *rhlA-gfp* reporter and measured fluorescence at 18 h. Fluorescence per cell is reported for clinical isolates (dark bars) with PAO1 (red), PAO1 Δ*lasR*, and PAO1 ΔRRR (blue) shown for comparison. Isolate E183, which demonstrated wild-type function, is also included. Growth with AiiA lactonase (light bars) abrogated the fluorescence in most cases. (D) Temporal profile of RhlR activation of the *rhlA-gfp* reporter. Strains E42 (closed circles), E131 (closed triangles), and E165 (open circles) are shown. PAO1 (closed squares) is shown in red, and PAO1 Δ*lasR* (inverted triangles) is shown in blue. In all panels, error bars show standard errors for results from four independent measurements. *, a statistically significant decrease (*P* < 0.05) in production of elastase (A), pyocyanin (B), or GFP (C) in the presence of AiiA, compared to the buffer control (two-tailed *t* test).

In agreement with prior work (19), our results showed that many LasR mutant CF isolates produce QS-regulated extracellular products, like elastase and pyocyanin, as well as C_4_-HSL, at levels sufficient for RhlR to activate gene expression in strain PAO1. We therefore hypothesized that RhlR signaling was independent of LasR in many isolates, such that production of some extracellular factors would still be controlled by QS. To test whether RhlR regulates elastase and pyocyanin in LasR mutant isolates, we grew several mutants in the presence of AiiA (20 µg/ml), an AHL-degrading lactonase purified from *Bacillus thuringiensis* (42). Neither C_4_- nor 3OC_12_-HSL was detectable in cultures with AiiA (see Table S3 in the supplemental material). The effect of AiiA on elastase and pyocyanin production was quite variable: AiiA lowered either elastase or pyocyanin levels, or both, in cultures of some isolates (e.g., E94, E97, E183) (Fig. 2A and B). However, in other isolates, neither elastase nor pyocyanin production was affected by AiiA. Yet other isolates had modest responses. Paradoxically, some isolates produced more elastase (for example, E120) or pyocyanin (for example, E106) in the presence of AiiA than in its absence. These results suggest that activation of QS-dependent factors in some CF LasR-null mutants is maintained by RhlR-C_4_-HSL, consistent with prior reports that RhlR can regulate these factors (36). In other isolates, however, there was complete decoupling of secreted product gene transcription from AHL quorum sensing.

To further test the hypothesis that the RhlR-RhlI system compensates for the loss of LasR in some isolates, we transformed isolates with a reporter plasmid containing *gfp* fused to the RhlR-dependent PAO1 *rhlA* promoter and grew transformants in the presence or absence of AiiA lactonase (Fig. 2C). Several of the transformants exhibited GFP fluorescence above background, and for four (E33, E89, E104, and E131) fluorescence exceeded that of strain PAO1. In all cases, GFP production was reduced substantially by the addition of AiiA. This result confirmed that in at least some isolates RhlR functions in the absence of LasR. In these isolates, RhlR functions in a cell-density-dependent manner, consistent with a role as quorum-sensing transcriptional regulator (Fig. 2D). This finding is different than findings from previous reports of LasR-independent RhlR activation (36, 40, 43), where RhlR was activated in late stationary phase. Together, these results suggest that RhlR, in some CF *P. aeruginosa* isolates, has replaced LasR as the primary AHL QS regulator.

## DISCUSSION

The emergence of LasR variants in CF and other chronic infections (4, 11, 44) presents an ecological and medical conundrum. It is not clear what benefits are conferred to bacteria with *lasR* mutations. It has been shown that *P. aeruginosa* LasR variants (presumed to be mutants) are associated with worsening CF lung disease, but a causal relationship has not been established (18, 45). In previous studies, isolates with *lasR* single-nucleotide substitutions in comparison with laboratory strains were generally thought to be LasR-null mutants. However, the model of the *P. aeruginosa* QS hierarchy that is used to predict the functional consequences of these coding changes, in which LasR is the master regulator, was based on studies with laboratory strains (46). We made use of a large collection of *P. aeruginosa* clinical isolates with *lasR* coding variations. As previously reported, a significant percentage of isolates had nonsynonymous *lasR* mutations in comparison to laboratory strains. However, we found greater complexity in QS regulation than anticipated. A substantial percentage of the LasR variants we tested retained LasR functionality, as measured by the ability to activate transcription of a reporter gene in *E. coli* or *P. aeruginosa* (Fig. 1B and C). We also found that in some LasR-null mutants that the RhlR-RhlI system was active and could overcome LasR defects in a way not exhibited by laboratory strains like PAO1, which has a QS hierarchy in which RhlR function, except under specific circumstances, depends on LasR (46).

Our data highlight the complexity of quorum sensing in *P. aeruginosa* in a diverse, naturally occurring isolate collection and suggest that a simple view of these *lasR* variants as quorum-sensing null mutants is inadequate to understand the biological consequences of these mutations. We have described two mechanisms by which quorum sensing is functional in our collection of *P. aeruginosa* isolates from patients with cystic fibrosis: first, polypepide sequence variants of LasR may not be completely inactive. The translated protein may retain the ability to activate transcription of QS-controlled genes in response to 3OC_12_-HSL. In all of the cases of this type, the activating concentration of 3OC_12_-HSL was similar to that required for the wild-type LasR, and we identified no LasR variant that could function in a signal-independent manner. In a diverse infecting population of signal-producing and signal-nonproducing cells, these LasR variants would easily be activated by signal produced by the population (19, 47) and might result in a wild-type LasR phenotype.

Second, RhlR can be functional in a LasR-independent manner, a phenomenon we observed in several isolates and in contrast to the laboratory strain PAO1. QS in *P. aeruginosa* has been described as a cascade hierarchy in which LasR activation precedes activation of RhlR and the non-AHL QS regulator PqsR (46), in part because in laboratory strains LasR inactivation downregulates these other QS systems. Even in these laboratory strains, some mechanisms have been described by which RhlR can function in a LasR-independent manner. For example, RhlR can be activated in LasR-null mutants under conditions of stress or activation of the stringent response (43), but such a state takes days in laboratory culture. Similarly, production of 2-alkyl-quinolone signals can activate the RhlR-I system in stationary phase (38) in some circumstances. We have found that these constraints are not present in our RhlR-I-active CF isolates.

RhlR in our clinical isolates was active in rich media and without the delay described for LasR-null laboratory strains (Fig. 2D), suggesting an alternative mechanism by which this QS regulator is functional. One possible mechanism for LasR-independent RhlR function involves PqsE (40, 48), although this pathway has not been elucidated (41, 49). This is a possibility in some of the isolates we tested in which quinolone signals and C_4_-HSL levels were increased compared to PAO1, such as E92 (Fig. 1E to G). However, it cannot account for the phenomenon in all of the isolates, as there are some isolates that have low levels of quinolone signals (e.g., E37). It is also formally possible that LasR does not always regulate RhlR in environmental strains of *P. aeruginosa*, although we do not have data to support this idea.

LasR mutants have been reported to emerge in other chronic infections, such as burns and diabetic wounds (11, 50). Our results did not reveal why LasR mutants emerge in chronic infections such as cystic fibrosis, but they do offer some clues. The preserved ability of different *P. aeruginosa* isolates to produce QS-regulated factors, such as elastase and phenazines in the absence of a functional LasR protein, supports the idea that these LasR mutants might gain a fitness advantage over the wild type (23, 24, 51) but still require the production of certain secreted products. In this model, the metabolic benefits of a *lasR* mutation outweigh any potential costs of losing the production of LasR-regulated factors, such as elastase. However, we found that in many isolates activities usually considered to be LasR dependent can be produced through other means, such as via RhlR directly, or through activation of RhlR via quinolone signaling molecules PQS, HHQ, or both (38).

In the setting of a chronic infection, many LasR-regulated genes are unlikely to be germane to survival, so inactivation of LasR could represent a substantial lifting of metabolic costs to an individual bacterium. Because there are mechanisms by which QS-regulated gene products continue to be produced in these LasR mutants, the bacteria are simply better adapted to the chronic infection.

The complexity of the QS system revealed by these isolates has significant implications for therapeutics directed at *P. aeruginosa* infections. The potential therapeutic benefit of using QS inhibitors in CF and similar chronic infections has been questioned, because LasR coding variants, which were presumed to signify inactive QS, were so frequently observed in these populations. Contrary to these assumptions, our data suggest that QS remains active in *P. aeruginosa* isolated from chronic CF infections. Many isolates previously thought to be LasR-null have functional LasR polypeptides with coding variations, and others have recruited the RhlR-RhlI system to activate genes usually considered to be QS dependent. We are currently exploring the genetic changes that accumulate in clinical *P. aeruginosa* isolates over time that might enable this plasticity in QS gene regulation. Our results further suggest that QS often remains functional despite mutations in *lasR.* Thus, broad-range QS inhibitors, targeting both the LasR-I and RhlR-I signaling systems, might be beneficial for chronic infections, such as those in the CF lung.

## MATERIALS AND METHODS

### Bacterial isolates, plasmids, and primers.

The strains, bacterial isolates, plasmids, and primers used are listed in Table S4 in the supplemental material. The clinical isolates were obtained in a multicenter study of Early *P. aeruginosa* Infection in Children with CF (the EPIC Observational Study). Patients in this study were aged 5 to 12 years, and the collection is composed of oropharyngeal and sputum isolates. Details regarding this collection of isolates and the study have been published previously (33). This study was approved by the Institutional Review Board (IRB) at Seattle Children’s Hospital.

### Quorum-sensing signal measurements.

We extracted AHLs from cultures of all *P. aeruginosa* isolates after 18 h of growth in morpholinopropanesulfonic acid (MOPS)-buffered Luria-Bertani (LB) broth (52). The starting culture had a density (OD_600_) of 0.025. Bacteria were grown with shaking (225 rpm) at 37°C in 18-mm test tubes containing 4 ml of buffered LB broth. AHLs were extracted with ethyl acetate as previously described (53). We used bioassays to measure C_4_-HSL and 3OC_12_-HSL in the ethyl acetate extracts, as described elsewhere, using *E. coli* DH5α containing pJN105L and pSC11 (for 3OC_12_-HSL) or pECP61.5 (for C_4_-HSL) (32, 54, 55). The bioassay strains and plasmids are listed in Table S4 in the supplemental material. Measurements of HHQ and PQS were performed by liquid chromatography-mass spectrometry analyses directly from culture supernatants, with tetradeutero-PQS and tetradeutero-HHQ as internal standards, as previously described (56, 57). Data are mean results with ranges for biological replicates.

### RhlR and LasR activity.

We constructed *lasI-gfp* (for LasR activity) and *rhlA-gfp* (for RhlR activity) fusions in pPROBE-GT (Gm^r^) (see Table S1 in the supplemental material) (58). We prepared electrocompetent cells from overnight cultures of each isolate using 300 mM sucrose and transformed cells by electroporation (59). Transformants were obtained by selective plating and confirmed by PCR. Transformants were grown as described above. GFP fluorescence of transformants was measured by using a Tecan fluorimeter. Growth rates of the various isolates differed substantially, and the 18-h time point ensured all were in stationary phase. PAO1 transformed with either plasmid was used as a control, and experiments were performed in triplicate.

### Protease and pyocyanin measurements.

To determine protease activity and pyocyanin production, we used 18-h LB broth cultures obtained as described above. Protease activity was measured with a fluorescent protease activity kit (Thermo Scientific) (47). We extracted pyocyanin from 18-h cultures as previously described (60). Briefly, cells were grown for 18 h in King’s A broth (61), and 4 ml whole culture was extracted with 2 ml chloroform. The organic layer was removed and reextracted with 0.2 N HCl. We measured absorbance of the aqueous layer at 520 nM.

### AiiA lactonase treatment.

We added 20 µg/ml purified AiiA lactonase (42) to cultures starting at an OD_600_ of 0.01, after overnight growth without lactonase in MOPS-buffered LB broth. Protease, pyocyanin, and AHLs were measured under the same growth conditions, as described above. For control cultures, we added the AiiA purification buffer only.

## SUPPLEMENTAL MATERIAL

Table S1  *lasR* sequence characterization of all 205 isolates in this study Table S1, PDF file, 0.04 MB

Table S2  Association between LasR function, colony morphology, and growth with nitrate Table S2, PDF file, 0.01 MB

Table S3  Effect of AiiA treatment on measured QS signal concentrations Table S3, PDF file, 0.01 MB

Table S4  Bacterial strains, plasmids, and primers used in this study Table S4, PDF file, 0.04 MB
