# Supplementary material for: LasR Variant Cystic Fibrosis Isolates Reveal an Adaptable Quorum-Sensing Hierarchy in Pseudomonas aeruginosa
Source: mBio. 2016 Oct 4;7(5):e01513-16. doi: 10.1128/mBio.01513-16 (PMC5050340; doi:10.1128/mBio.01513-16)
Supplement: Table S1 — lasR sequence characterization of all 205 isolates in this study [file mbo005163010st1.pdf]

Table S1. *lasR* sequence characterization of all 205 isolates in this study

Isolate designation	*lasR* associated mutation	Nucleotide change	Amino acid change
**E01**	whole gene deletion		
**E02**	whole gene deletion		
**E03**	whole gene deletion		
**E04**	whole gene deletion		
**E05**	whole gene deletion		
**E06**	whole gene deletion		
**E07**	whole gene deletion		
**E08**	whole gene deletion		
**E09**	whole gene deletion		
**E10**	whole gene deletion		
**E11**	whole gene deletion		
**E12**	whole gene deletion		
**E13**	whole gene deletion		
**E14**	whole gene deletion		
**E15**	whole gene deletion		
**E16**	whole gene deletion		
**E17**	whole gene deletion		
**E18**	whole gene deletion		
**E19**	whole gene deletion		
**E20**	whole gene deletion		
**E21**	whole gene deletion		
**E22**	whole gene deletion		
**E23**	whole gene deletion		
**E24**	whole gene deletion		
**E25**	whole gene deletion		
**E26**	whole gene deletion		
**E27**	whole gene deletion		
**E28**	whole gene deletion		
**E29**	whole gene deletion		
**E30**	Non-synonymous	G377A	G126D
**E31**	Non-synonymous	C133T	Q45Stop
**E32**	Non-synonymous	C649T	R217W
**E33**	Non-synonymous	G61A	A21T
**E34**	Non-synonymous	C292T	Q98Stop
**E35**	Insertion	IS Element @ 563	IS Element
**E36**	Deletion	13 bp deletion @ 493 (-CTGGCCTTCGAAC)	Frameshift
**E37**	Non-synonymous	G588T	E196D
**E38**	Deletion	11 bp deletion @ 98 (-CGAAGATCCTG)	Frameshift
**E39**	Non-synonymous	G541A	E181K
**E40**	Non-synonymous	C670T	R224C
**E41**	Non-synonymous	G31T	E11Stop
**E42**	Non-synonymous	G671A	R224H
**E43**	Deletion	1 bp deletion @ 407 (-A)	Frameshift
**E44**	Non-synonymous	C221T	P74L
**E45**	Non-synonymous	C70T	Q24Stop
**E46**	Deletion	3 bp deletion @ 349 (-CCG)	1 aa deletion (-P117)

**E47**	Insertion	1 bp insertion @ 342 (+T)	Frameshift
**E48**	Non-synonymous	C181A	R61S
**E49**	Non-synonymous	G56A	W19Stop
**E50**	Non-synonymous	C564A	C188Stop
**E51**	Wildtype	Wildtype	Wildtype
**E52**	Non-synonymous	C706T	L236F
**E53**	Insertion	IS Element @ 129	IS Element
**E54**	Non-synonymous	C350T	P117L
**E55**	Non-synonymous	G386A	S129N
**E56**	Non-synonymous	G338A	G113E
**E57**	Non-synonymous	G307T	E103Stop
**E58**	Non-synonymous	C692T	A231V
**E59**	Non-synonymous	A625G	N209D
**E60**	Non-synonymous	T68G	L23R
**E61**	Non-synonymous	T707G	L236R
**E62**	Non-synonymous	A467T	D156V
**E63**	Non-synonymous	A653G	K218R
**E64**	Deletion	5 bp deletion @ 445 (-CCGAC)	Frameshift
**E65**	Non-synonymous	G616A	A206T
**E66**	Non-synonymous	C221A	P74Q
**E67**	Non-synonymous	G179A	W60Stop
**E68**	Non-synonymous	T227G	V76G
**E69**	Non-synonymous	C611A	S204Stop
**E70**	Non-synonymous	C631A	H211N
**E71**	Non-synonymous	T592C	S198P
**E72**	Non-synonymous	C556T	Q186Stop
**E73**	Non-synonymous	G622A	V208M
**E74**	Non-synonymous	G676	V226I
**E75**	Non-synonymous	C670A	R224S
**E76**	Non-synonymous	G184C	E62Stop
**E77**	Non-synonymous	G571A	G191S
**E78**	Non-synonymous	C665T	T222I
**E79**	Insertion	IS Element @ 130	IS Element
**E80**	Deletion	4 bp deletion @ 319 bp (-GGCC)	Frameshift
**E81**	Deletion	13 bp deletion @ 389 (-TCAGCGTGGAAGC)	Frameshift
**E82**	Insertion	IS Element @ 630	IS Element
**E83**	Non-synonymous	C413T	A138V
**E84**	Non-synonymous	G704C	G235A
**E85**	Insertion	IS Element @ 143	IS Element
**E86**	Deletion	11 bp deletion @ 97 (-TCGAAGATCCT)	Frameshift
**E87**	Insertion	IS Element @ 241	IS Element
**E88**	Non-synonymous	C692A	A231D
**E89**	Non-synonymous	G543C	E181D
**E90**	Deletion	1 bp deletion @ 170 (-C)	Frameshift
**E91**	Insertion	IS Element @ 705	IS Element
**E92**	Non-synonymous	G490T	G164Stop
**E93**	Insertion	IS Element @ 140	IS Element
**E94**	Insertion	IS Element @ 139	IS Element
**E95**	Non-synonymous	G671T	R224L

**E96**	Insertion	IS Element @ 266	IS Element
**E97**	Insertion	IS Element @ 600	IS Element
**E98**	Insertion	1 bp insertion @ 415 (+C)	Frameshift
**E99**	Non-synonymous	T629G	F210C
**E100**	Non-synonymous	A641G	N214S
**E101**	Deletion	1 bp deletion @ 422 (-A)	Frameshift
**E102**	Non-synonymous	C646T	R216W
**E103**	Non-synonymous	T68C	L23P
**E104**	Non-synonymous	A532G	T178A
**E105**	Deletion	2 bp deletion @ 2 (-TG)	Frameshift
**E106**	Non-synonymous	C239T	T80I
**E107**	Non-synonymous	G539A	R180Q
**E108**	Non-synonymous	C355T	H119Y
**E109**	Non-synonymous	A223C	T75P
**E110**	Non-synonymous	G25A	E9K
**E111**	Non-synonymous	A542G	E181G
**E112**	Insertion	IS Element @ 716	IS Element
**E113**	Non-synonymous	T55C	W19R
**E114**	Insertion	1 bp insertion @ 452 (+C)	Frameshift
**E115**	Non-synonymous	C220T	P74S
**E116**	Non-synonymous	C683A	A228D
**E117**	Non-synonymous	A712G	T238A
**E118**	Insertion	1 bp insertion @ 449 (+C)	Frameshift
**E119**	Insertion	IS Element @ 463	IS Element
**E120**	Insertion	8 bp insertion @ 639 (+CATATGGG)	Frameshift
**E121**	Non-synonymous	G376T	G126C
**E122**	Non-synonymous	G92T	G31V
**E123**	Non-synonymous	C617T	A206V
**E124**	Non-synonymous	G376A	G126S
**E125**	Non-synonymous	C280T	Q94Stop
**E126**	Non-synonymous	G654T	K218N
**E127**	Non-synonymous	C564G	C188W
**E128**	Insertion	IS Element @ 551	IS Element
**E129**	Non-synonymous	C149T	A50V
**E130**	Non-synonymous	T662A	V221E
**E131**	Non-synonymous	A580G	S194G
**E132**	Insertion	1 bp insertion @ 173 (+G)	Frameshift
**E133**	Non-synonymous	G313A	A105T
**E134**	Non-synonymous	C683T	A228V
**E135**	Deletion	30 bp deletion @ 604 (-AACTGCTCGGAAGCCAATGTGAACTTCCAT)	10 aa deletion (-NCSEANVNFH 201-212)
**E136**	Deletion	11 bp deletion @ 423 (-CCGTTTCATGG)	Frameshift
**E137**	Non-synonymous	G455A	W152Stop
**E138**	Deletion	14 bp deletion @ 565 (-GCCATCGGCAAGAC)	Frameshift
**E139**	Non-synonymous	T178G	W60G
**E140**	Deletion	1 bp deletion @ 564 (-C)	Frameshift
**E141**	Insertion	42 bp insertion @ 141 (+GAAGATCCTGTTCGGCCTGTTGCCTAAGGACAGCCAGGACTA)	Stop + 7 aa insertion @ 47 (+*KILFGLLPKDSQD)
**E142**	Non-synonymous	C219A	D73E
**E143**	Non-synonymous	A605T	N202I
**E144**	Non-synonymous	G585A	W195Stop

**E145**	Non-synonymous	C219G	D73E
**E146**	Non-synonymous	G638A	G213E
**E147**	Deletion	2 bp deletion @ 562 (-TG)	Frameshift
**E148**	Non-synonymous	C165A	N55K
**E149**	Deletion	1 bp deletion @ 337 (-G)	Frameshift
**E150**	Non-synonymous	G377C	G126A
**E151**	Non-synonymous	G199C	A67P
**E152**	Non-synonymous	G182A	R61H
**E153**	Insertion	IS Element @ 647	IS Element
**E154**	Non-synonymous	C350T	P117L
**E155**	Non-synonymous	T559C	W187R
**E156**	Non-synonymous	G226T	V76F
**E157**	Deletion	1 bp deletion @ 414 (-C)	Frameshift
**E158**	Non-synonymous	G661A	V221M
**E159**	Non-synonymous	G264A	W88Stop
**E160**	Insertion	3 bp insertion @ 334 (+TTG)	1 aa insertion @ 112 (+L)
**E161**	Insertion	1 bp insertion @ 452 (+A)	Frameshift
**E162**	Non-synonymous	C529G	L177V
**E163**	Non-synonymous	G691A	A231T
**E164**	Non-synonymous	G415T	E139Stop
**E165**	Non-synonymous	G703A	G235S
**E166**	Non-synonymous	G654C	K218N
**E167**	Deletion	1 bp deletion @ 339 (-G)	Frameshift
**E168**	Non-synonymous	T707C	L236P
**E169**	Non-synonymous	C437A	S146Stop
**E170**	Insertion	24 bp insertion @ 355 (+GTGTATGGGCTGACCATGCCGCTG)	8 aa insertion @ 119 (+VYGLTMPL)
**E171**	Non-synonymous	T316C	S106P
**E172**	Non-synonymous	G57A	W19Stop
**E173**	Deletion	10 bp deletion @ 583 (-TGGGAGATAT)	Frameshift
**E174**	Non-synonymous	T583A	W195R
**E175**	Deletion	1 bp deletion @ 663 (-G)	Frameshift
**E176**	Deletion	10 bp deletion @ 627 (-CTTCCATATG)	Frameshift
**E177**	Non-synonymous	G386A	S129N
**E178**	Non-synonymous	G319C	A107P
**E179**	Non-synonymous	G560A	W187Stop
**E180**	Non-synonymous	G547A	E183K
**E181**	Insertion	IS Element @ 561	IS Element
**E182**	Insertion	4 bp insertion @ 580 (+GACC)	Frameshift
**E183**	Non-synonymous	C478T	Q160Stop
**E184**	Non-synonymous	G337C	G113R
**E185**	Non-synonymous	C668A	S223Y
**E186**	Non-synonymous	C314T	A105V
**E187**	Insertion	IS Element @ 239	IS Element
**E188**	Non-synonymous	T452C	L151P
**E189**	WT		
**E190**	WT		
**E191**	WT		
**E192**	WT		
**E193**	WT		

**E194**	WT		
**E195**	WT		
**E196**	WT		
**E197**	WT		
**E198**	WT		
**E199**	Insertion	1 bp insertion @ 281 (+C)	Frameshift
**E200**	Non-synonymous	G217A	D73N
**E201**	Non-synonymous	T607A	C203S
**E202**	Insertion	IS Element @ 467	IS Element
**E203**	Non-synonymous	G682C	A228P
**E204**	Non-synonymous	C254T	P85L
**E205**	Deletion	3 bp deletion @ 637 (-GGA) + A640G	1 aa deletion (-G213) + N214D
