# Supplementary material for: LasR Variant Cystic Fibrosis Isolates Reveal an Adaptable Quorum-Sensing Hierarchy in Pseudomonas aeruginosa
Source: mBio. 2016 Oct 4;7(5):e01513-16. doi: 10.1128/mBio.01513-16 (PMC5050340; doi:10.1128/mBio.01513-16)
Supplement: Table S2 — Association between LasR function, colony morphology, and growth with nitrate [file mbo005163010st2.pdf]

Table S2. Association between LasR function, colony morphology, and growth with nitrate[a]

Phenotype	Genotype: Wild type LasR (n=1996)	Genotype: Inactivating mutation (n=268)
Lysis, sheen, or both[b]	171 (8.6[c])	190 (70.9)
Neither lysis nor sheen	1825 (91.4)	78 (29.1)
NO_3 growth advantage[d]	170 (8.5)	69 (25.7)
No NO_3 growth advantage	1826 (91.5)	199 (74.3)
Lysis, sheen, and NO_3 growth advantage[e]	18 (0.9)	44 (16.4)

[a]Isolates for which we could not deterimine LasR functionality are not included.

[b]Colony morphology of isolates grown on LB agar as described in Ref. 3.

[c]Data in parentheses represents the percent of isolates with specific phenotype.

[d]Growth advantage with nitrate (NO_3) supplementation as described in Ref. 18.

[e]Isolates that display lysis and sheen and have increased growth with nitrate.
