# Supplementary material for: LasR Variant Cystic Fibrosis Isolates Reveal an Adaptable Quorum-Sensing Hierarchy in Pseudomonas aeruginosa
Source: mBio. 2016 Oct 4;7(5):e01513-16. doi: 10.1128/mBio.01513-16 (PMC5050340; doi:10.1128/mBio.01513-16)
Supplement: Table S3 — Effect of AiiA treatment on measured QS signal concentrations [file mbo005163010st3.pdf]

Table S3. Effect of AiiA treatment on measured signal concentrations

	$3OC_{12}$-HSL (μM)		C_4-HSL (μM)	
Strain or isolate	- AiiA	+ AiiA	- AiiA	+ AiiA
PAO1	2.80	0.044	4.60	< 0.005
PAO1 Δ*lasR*	< 0.005	< 0.005	0.47	< 0.005
E57	0.026	< 0.005	5.15	< 0.005
E91	0.011	< 0.005	5.53	< 0.005
E113	0.045	< 0.005	7.27	0.028
E188	0.018	< 0.005	6.06	< 0.005
E202	0.013	< 0.005	12.71	< 0.005
