# Supplementary material for: LasR Variant Cystic Fibrosis Isolates Reveal an Adaptable Quorum-Sensing Hierarchy in Pseudomonas aeruginosa
Source: mBio. 2016 Oct 4;7(5):e01513-16. doi: 10.1128/mBio.01513-16 (PMC5050340; doi:10.1128/mBio.01513-16)
Supplement: Table S4 — Bacterial strains, plasmids, and primers used in this study [file mbo005163010st4.pdf]

Table S4. Bacterial strains, plasmids, and primers used in this study.

| Bacterial strain or plasmid | Description | Reference or source |
|---|---|---|
| *P. aeruginosa* strains | | |
| PAO1 | Wild type strain | (1) |
| PAO1 Δ*lasR* | PAO1 containing an unmarked, in-frame *lasR* deletion | (2) |
| PAO1 Δ*lasR* Δ*rhlR* Δ*qscR* | *lasR, rhlR, qscR triple* mutant in PAO1 | (3) |
| Other strains | | |
| *E. coli* DH5α | $F^-$ Φ80*lac*ZΔM15 Δ(*lac*ZYA-*arg*F) U169 *rec*A1 *end*A1 *hsd*R17($r_k^-$, $m_k^+$) *pho*A*sup*E44 *thi*-1 *gyr*A96 *rel*A1 $λ^-$ | Invitrogen |
| Plasmids | | |
| pSC11 | Broad-host-range *lasI-lacZ* reporter, $Ap^r$ | (4) |
| pJN105L | Arabinose inducible *lasR* in pJN105, $Gm^r$ | (5) |
| pECP61.5 | Contains an *rhlA-lacZ* translation fusion and IPTG-inducible *rhlR* , $Ap^r$ | (6) |
| pPROBE-GT | Broad-host-range pVS1/p15a GFP reporter, $Gm^r$ | (7) |
| pJF01 | pPROBE-GT with -1 through-501 5' region of *rhlA* inserted with HindIII and BamHI, $Gm^r$ | This work |
| pBS351 | pPROBE-GT with -1 through-501 5' region of *lasI* inserted with HindIII and BamHI, $Gm^r$ | This work |

| | |
|---|---|
| *Primers* | |
| lasR.cloning.EcoR1 F | 5' – TTTTTGAATTCTAGCGCTATGGCCTTGGTT – 3' |
| lasR.cloning.Xba1 R | 5' – AAAAATCTAGAGCAAGATCAGAGAGTAATAAGACCC – 3' |
| rhlA.GFPcloning.HindIII F | 5' – TTTTTAAGCTTGCATGCGAGGCCTGCGAA – 3' |
| rhlA.GFPcloning.BamHI R | 5' – AAAAAAGGATCCTCTAGAAACCGATACCAACAGACTTTCGC – 3' |
| GFP.QC R | 5' – TTCTTTCCTGTACATAACCTTCGGGCA – 3' |

## REFERENCES


1. **Stover CK, Pham XQ, Erwin AL, Mizoguchi SD, Warrener P, Hickey MJ, Brinkman FS, Hufnagle WO, Kowalik DJ, Lagrou M, Garber RL, Goltry L, Tolentino E, Westbrock-Wadman S, Yuan Y, Brody LL, Coulter SN, Folger KR, Kas A, Larbig K, Lim R, Smith K, Spencer D, Wong GK, Wu Z, Paulsen IT, Reizer J, Saier MH, Hancock RE, Lory S, Olson MV.** 2000. Complete genome sequence of *Pseudomonas aeruginosa* PAO1, an opportunistic pathogen. Nature **406:**959-964.
2. **Wang M, Schaefer AL, Dandekar AA, Greenberg EP.** 2015. Quorum sensing and policing of *Pseudomonas aeruginosa* social cheaters. Proc Natl Acad Sci USA **112:**2187-2191.
3. **Chugani S, Greenberg EP.** 2010. LuxR homolog-independent gene regulation by acyl-homoserine lactones in *Pseudomonas aeruginosa*. Proc Natl Acad Sci USA **107:**10673-10678.
4. **Chugani SA, Whiteley M, Lee KM, D'Argenio D, Manoil C, Greenberg EP.** 2001. QscR, a modulator of quorum-sensing signal synthesis and virulence in *Pseudomonas aeruginosa*. Proc Natl Acad Sci USA **98:**2752-2757.
5. **Lee JH, Lequette Y, Greenberg EP.** 2006. Activity of purified QscR, a *Pseudomonas aeruginosa* orphan quorum-sensing transcription factor. Mol Microbiol **59:**602-609.
6. **Pearson JP, Pesci EC, Iglewski BH.** 1997. Roles of *Pseudomonas aeruginosa las* and *rhl* quorum-sensing systems in control of elastase and rhamnolipid biosynthesis genes. J Bacteriol **179:**5756-5767.
7. **Miller WG, Leveau JH, Lindow SE.** 2000. Improved *gfp* and *inaZ* broad-host-range promoter-probe vectors. Mol Plant Microbe Interact **13:**1243-1250.
